# Species Delimitation Using Genomic Data: Options and Limitations

**DOI:** 10.1111/mec.17717

**Published:** 2025-03-03

**Authors:** Bernhard Hausdorf

**Affiliations:** ^1^ Leibniz Institute for the Analysis of Biodiversity Change Hamburg Germany; ^2^ Universität Hamburg Hamburg Germany

**Keywords:** DNA taxonomy, isolation by distance, multispecies coalescent, population genetics, species delimitation, species validation

## Abstract

The most effective approaches for species discovery and species validation with genomic data remain underexplored. This study evaluates the merits and limitations of phylogenetic approaches based on the multispecies coalescent model and population genetic approaches for species discovery, i.e., species delimitation in the absence of prior knowledge, using genomic datasets from four well‐known radiations. Furthermore, it demonstrates how geographic data can be integrated with the genomic data for species validation, i.e., for testing primary species hypotheses. The multispecies coalescent model‐based approaches tr2 and soda resulted in high over‐splitting of species, low percentages of species delimited according to the current classification, and low percentages of individuals assigned to the same species as in the current classification across all four species complexes studied. Conversely, the species numbers were slightly underestimated based on the structure results. Although the proportion of species delimited according to the current classification and the proportion of individuals assigned to the same species as in the current classification in the classifications based on the structure results is approximately twice that of the classifications proposed by the multispecies coalescent model‐based approaches, it remains unsatisfactory. A slight over‐splitting of species into population groups can be corrected by species validation with isolation‐by‐distance tests if a sufficient number of populations have been sampled for each species. Sampling design is an essential step in any taxonomic study, as it has a significant impact on the delimitation of the species and the possibility of their validation.

## 1 Introduction

The significance of DNA‐based approaches for delimiting species has grown considerably over the last two decades. In the early stages of DNA‐based taxonomic studies, particularly the ‘DNA barcoding’ approach (Hebert et al. 2003), the majority of studies employed a single or limited number of markers, frequently comprising mitochondrial DNA fragments. This is an expedient and cost‐effective method for obtaining preliminary insights into the taxonomic structure of a group. This approach may prove effective in delimiting reproductively isolated species that have been separated for a sufficient period of time for lineage sorting to be complete following speciation. However, this approach is inadequate for resolving the taxonomy of groups that radiated in a short time span. The probability of sequences of conspecific individuals not forming a monophyletic group in gene trees is high when the time since the separation of these species is short (Neigel and Avise 1986). Furthermore, evolutionary studies have demonstrated that diverging groups of populations, which overlap geographically and remain distinct in areas of range contact or overlap and are therefore considered to be distinct species, are often not completely reproductively isolated as the biological species concept (Mayr 1963) envisions. Rather, hybrids can be produced that are fertile and can backcross with the parental species so that many genetic markers can be exchanged between diverging species over an extended period of time (Abbott et al. 2013; Coyne and Orr 2004; Hausdorf 2011; Mallet 2005, 2008; Wu 2001; Wu and Ting 2004). In fact, approximately 20% of the species examined in the literature were found to be paraphyletic or polyphyletic in mitochondrial gene trees in literature surveys (Funk and Omland 2003; Ross 2014). Incomplete lineage sorting and hybridization can only be identified through the analysis of multiple recombining markers.

The development of approaches such as reduced representation DNA sequencing (Andrews et al. 2016; Hohenlohe et al. 2021) has enabled the generation of large genomic multilocus datasets at reasonable costs. This has facilitated the detection of incomplete lineage sorting and hybridization in non‐model organisms. Nevertheless, the delimitation of closely related species remains a challenging undertaking. The evolution of species boundaries represents the transition between population genetic processes within populations and population groups (or tokogenetic relationships; Hennig 1966) and phylogenetic relationships between independently evolving taxa. Very different approaches are employed for the analysis of population genetic structure, which is characterised by gene flow and changes in allele frequencies, and the investigation of phylogenetic relationships among species, the species tree. The latter utilises the information present in gene trees, which elucidate the relationships among alleles of a gene. Approaches that are rooted in either of these areas are applied for species delimitation. The preference for specific methods is often less guided by the efficiency of the applied methods than by the phylogenetic or population genetic background of the researchers.

From a phylogenetic standpoint, a significant advancement beyond the incorrect equation of a gene tree with the species tree was the probabilistic modelling of the relationship between gene trees and the species history within the multispecies coalescent (MSC) framework, which considers lineage sorting in an appropriate manner (Fujisawa et al. 2016; Fujita et al. 2012; Knowles and Carstens 2007; Rabiee and Mirarab 2020; Rannala and Yang 2003; Yang and Rannala 2014). However, the MSC model used for species delimitation assumes that there is neutral random coalescence without structure within species (i.e., random mating) and no gene flow after species divergence (Fujisawa et al. 2016; Rabiee and Mirarab 2020; Rannala and Yang 2003).

Given the high level of introgression found in some radiations, it may be useful to compute a network to get a first idea of how tree‐like the relationships between species in the study group are.

Population genetic approaches for inferring population structure such as structure (Pritchard et al. 2000) estimate population structure and individual ancestry by modelling Hardy–Weinberg equilibrium within populations. These approaches explicitly consider admixture and gene flow. It has been demonstrated that such methodologies may also prove beneficial for species delimitation, provided that appropriate sampling is employed (Bamberger et al. 2022; Hausdorf and Hennig 2010; Rittmeyer and Austin 2012; Sauer and Hausdorf 2012; Shaffer and Thomson 2007). In the population genetic approach, primary species hypotheses are operationally based on genotypic clusters corresponding to ancestral populations. It should be noted that these approaches do not directly provide a classification of individuals into species. However, their results can be transformed into primary species hypotheses by considering the ancestral populations from which the majority of the examined individuals' genomes are derived (Bamberger et al. 2022).

Species delimitation based on a single kind of evidence may miss important aspects of the speciation process. Therefore, it is recommended to combine genetic data with other kinds of data (e.g., morphological, ecological, geographical data) in integrative approaches for species delimitation (Padial et al. 2010; Sauer and Hausdorf 2012; Schlick‐Steiner et al. 2010). However, methods that can use different kinds of data for species discovery or species validation are still in their infancy (Edwards and Knowles 2014; Guillot et al. 2012; Hausdorf and Hennig 2020; Solís‐Lemus et al. 2015).

This study assesses the merits and limitations of phylogenetic approaches based on the MSC model and a population genetic approach for inferring population structure for species discovery, i.e. species delimitation in the absence of prior knowledge, based on genomic datasets including Metazoa‐level universal single‐copy orthologs (USCOs) of four well‐known radiations: *Anopheles gambiae* complex mosquitoes (Fontaine et al. 2015), *Drosophila nasuta* species complex fruit flies (Mai et al. 2019), *Heliconius melpomene* complex butterflies (Martin et al. 2013), and Darwin's finches (Lamichhaney et al. 2015), which were compiled by Dietz et al. (2024). Dietz et al. (2024) showed that USCOs are genetically unlinked for practical purposes and a representative sample of a genome in terms of reciprocal distances between USCOs on a chromosome and of distribution across chromosomes and that they yield phylogenies that corresponded to those generated from whole genome data. With regard to species delimitation, Dietz et al. (2024) applied two MSC methods to the USCO datasets, which split most morphospecies in the *Anopheles* and *Drosophila* datasets into multiple species, whereas many morphospecies of Darwin's finches and some (but not all) subspecies of *Heliconius melpomene* were lumped. In complement to their analyses, I will use a population genetic approach to delimit primary species hypotheses with the USCO datasets of the four radiations. In a second step, geographic data are integrated with the genomic data for species validation, i.e. for testing the primary species hypotheses through the application of isolation‐by‐distance (IBD) tests proposed by Hausdorf and Hennig (2020). I will compare the results of this two‐step approach with the original classifications and the results of the MSC methods provided by Dietz et al. (2024), taking into account the assumptions underlying these methods. Based on these results and shortcomings of the species delimitation approaches, I will make recommendations for taxonomic studies.

## 2 Materials and Methods

### 2.1 Datasets

The Metazoa‐level USCOs datasets compiled by Dietz et al. (2024) from genome data of four radiations, the *Anopheles gambiae* complex (Fontaine et al. 2015), the *Drosophila nasuta* species complex (Mai et al. 2019), the *Heliconius melpomene* complex (Martin et al. 2013), and Darwin's finches (Lamichhaney et al. 2015), were employed in combination with various species delimitation approaches. Dietz et al. (2024) used three different methodologies to extract USCO nucleotide sequences from the reference genomes, with the objective of evaluating the data yield and the ability to resolve species‐level relationships. In the first approach, the exonic nucleotide sequences of USCOs were extracted from the assembled genomes with the busco program v. 4.0.6 (Manni et al. 2021; Simão et al. 2015) utilising the genome mode and the Metazoa dataset from OrthoDB v. 10 (Kriventseva et al. 2019), henceforth referred to as the BUSCO dataset. In the second approach, Orthograph v. 0.7.1 (Petersen et al. 2017) was employed in conjunction with hidden Markov models from OrthoDB v. 9 (Zdobnov et al. 2017), which will henceforth be referred to as the OrthoDB9 dataset. The third approach was identical to the second, with the exception that OrthoDB v. 10 (https://busco.ezlab.org/busco_v4_data.html) was used in the following text referred to as the OrthoDB10 dataset. Further details can be found in Dietz et al. (2024).

Dietz et al. (2024) extracted USCOs with the three approaches from one selected fully assembled and annotated genome per study group. Subsequently, each gene sequence was employed as a reference against which the raw reads of all individuals of each dataset were mapped with bwa v. 2.1 (Li and Durbin 2009). Diploid consensus sequences, in which heterozygous sites were represented by an IUPAC ambiguity code, were generated with samtools v. 1.10 (Li et al. 2009) and bcftools v. 1.10.2 (https://github.com/samtools/bcftools). Dietz et al. (2024) conducted phylogenetic analyses based on the nucleotide sequence alignment of each individual USCO with iq‐tree v. 2.1.2 (Minh et al. 2020). The resulting trees were then employed as input for a MSC analysis with astral v. 5.6.1 (Zhang et al. 2018). All trees were rooted with the outgroup taxa used in the respective original studies from which the data were derived.

Dietz et al. (2024) extracted single‐nucleotide polymorphisms (SNPs) from the USCO nucleotide alignments of diploid consensus sequences with the software snp‐sites (Page et al. 2016), excluding low‐quality sites masked by the software bcftools with lowercase letters. All outgroup taxa were excluded from the multiple nucleotide sequence alignments. In the dataset of Darwin's finches, the divergent ingroup genus *Certhidea* was also excluded. For more details, see Dietz et al. (2024).

### 2.2 Species Discovery Approaches

I compared the performance of two methods based on the MSC model‐based programs and a population genetic method for delimiting primary species hypotheses without prior knowledge. The methods based on the MSC model assume that there is neutral random coalescence without structure within species (i.e., random mating across the whole range) and no gene flow after species divergence (Fujisawa et al. 2016; Rabiee and Mirarab 2020; Rannala and Yang 2003). Dietz et al. (2024) employed the MSC model‐based species delimitation approaches tr2 (Fujisawa et al. 2016) and soda v. 1.0.2 (Rabiee and Mirarab 2020) to delimit species based on the three different USCO datasets they had compiled for each of the four studied radiations. tr2 infers species boundaries from the distribution of rooted topologies of triplets of individuals and a guide tree, whereas soda uses the distribution of unrooted topologies of quartets of individuals to the same end. Only trees that contained all specimens could be used for tr2, as the program is unable to process gene trees that lack specimens. In contrast, all gene trees could be used for soda. I used the results of these methods reported by Dietz et al. (2024) here for a comparison with a species discovery approach based on a population genetic method.

The population genetic method structure (Pritchard et al. 2000) estimates population structure and individual ancestry by modelling Hardy–Weinberg equilibrium within populations and explicitly considers admixture (albeit without an explicit model of gene flow). Dietz et al. (2024) conducted a structure v. 2.3.4 analysis based on SNPs with a Markov chain Monte Carlo chain length of 50,000 (burn‐in of 20,000) and a range of values for the number of ancestral populations (*K*) from 1 to 10. The analysis was repeated 10 times for each value of *K*, and the result with the highest likelihood was selected. I transformed the proportions of individual ancestry estimated in these structure analyses into primary species hypotheses by assigning each individual to the cluster that corresponded to the reconstructed ancestral population from which the largest proportion of its genome was derived (see also Bamberger et al. 2022). Individuals with high levels of admixture are assigned to one of the species because this is necessary to be able to include them in the IBD tests (see below). If we did not include the hybrids in the IBD tests, we would ignore evidence against the species status of the primary species hypothesis and obtain biased results. This does not mean that the species status of individuals with high levels of admixture is equivalent to that of individuals with little or no admixture. In a discussion, they should be classified as hybrids.

The sensitivity of the species delimitation approach based on structure to the choice of the appropriate *K* was explored by comparing the primary species hypotheses derived from the results with the highest likelihoods for *K* (of the run with the overall highest likelihood) minus 1 and plus 1 with the classification based on the run with the overall highest likelihood using the BUSCO dataset.

### 2.3 Species Delimitation Parameters

The classification success of the various approaches is summarised in a number of parameters. structure is used for delimiting populations or closely related species. Therefore, outgroups and, in the case of Darwin's finches also the genus *Certhidea*, which is separated from the other groups by a deep split, were not considered in the structure analyses and the parameters. The number of individuals in the *Drosophila* and *Heliconius* datasets is lower than in Dietz et al. (2024; table 1) due to the exclusion of outgroup individuals ( *Drosophila immigrans* and *Heliconius pardalinus*, *H. hecale* and *H. ethilla*, respectively). In the Darwin's finches dataset, the numbers of species and individuals are lower than in Dietz et al. (2024; table 1) due to the exclusion of the *Certhidea* species ( *Certhidea olivacea* and *Certhidea fusca* ) from the structure analyses, in addition to the outgroups. The classifications used in the original studies were used here as references for comparisons because they were usually based also on additional (morphologically, behavioural, etc.) data and are the current state of the art. This does not mean that they were accepted as true. Rather, improvements of the current classifications will be suggested based on the analyses presented here in the Discussion. A species is considered to be delimited in congruence with the current classification only if the inferred species cluster contains exactly the individuals included in the species in the original publication. Individuals are deemed to be assigned to the same species as in the current classification also if the delimited cluster includes individuals of other species. Individuals of a single species can be counted as assigned to the same species as in the current classification only in a single delimited cluster, and only one species is counted in each delimited cluster. If a species is represented in more than one cluster, the highest number of individuals belonging to this species in a single cluster is counted.

### 2.4 Network Analyses

As introgression between closely related species is common, especially in radiations, a network may be a more appropriate representation of relationships between the species than a bifurcating tree, and it may indicate introgression between species. Neighbor‐Net (Bryant and Moulton 2004) networks are chosen here because they can also be constructed with large genomic datasets. The Neighbor‐Net networks were constructed based on shared allele distances (Bowcock et al. 1994) between individuals using splitstree4 v.4.18.3 (Huson and Bryant 2006). Shared allele distances were calculated based on SNP data using the function ‘alleledist’ of prabclus (Hennig and Hausdorf 2020), an add‐on package for the statistical software R (R Core Team 2022). The pairwise distance calculations were conducted without consideration of SNPs with missing data.

### 2.5 IBD Tests

Primary species hypotheses derived from the literature or from the structure results were assessed using the IBD tests proposed by Hausdorf and Hennig (2020). IBD tests investigate the hypothesis that the genetic distances between individuals belonging to two primary species hypotheses can be explained by IBD. The null hypothesis is that the genetic distances between individuals are not larger than expected based on their geographical distances and the relationship of genetic and geographical distances within the primary species hypotheses. Samples of at least one of the species of a pair from at least three different populations are necessary for an IBD test. The IBD tests were calculated using shared allele distances based on SNP data with the R package prabclus (Hennig and Hausdorf 2020).

## 3 Results

### 3.1 Species Discovery

**FIGURE 1:**
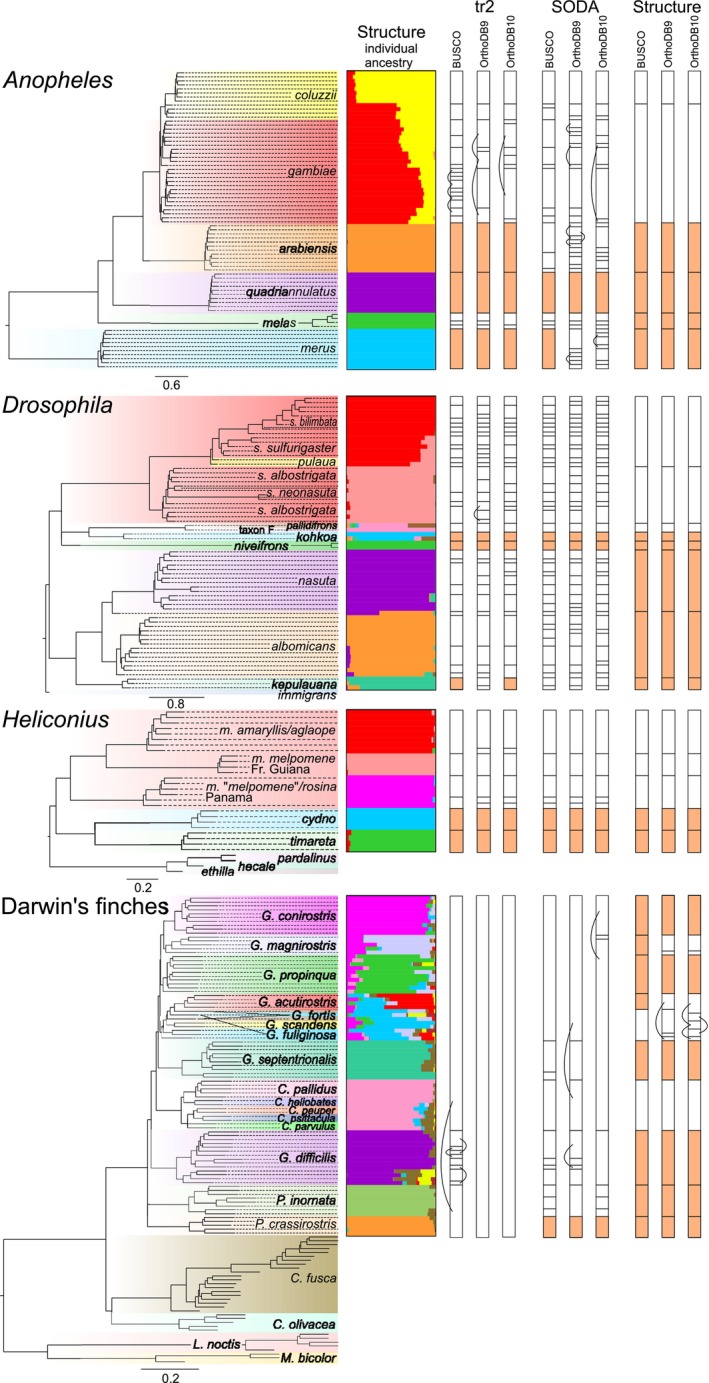
Species tree of the four radiations studied, structure results (individual ancestry) based on the BUSCO dataset and species classifications obtained using tr2, soda and derived from the structure results with the three different data assemblies. Background colour and labels on the tree indicate the species classifications in the original references. Coloured boxes in the species classification columns indicate that inferred clusters are congruent with species in the original classifications and curved lines connect partitions belonging together according to the respective classification but are separated by other partitions (partly modified from Dietz et al. 2024).

**FIGURE 2:**
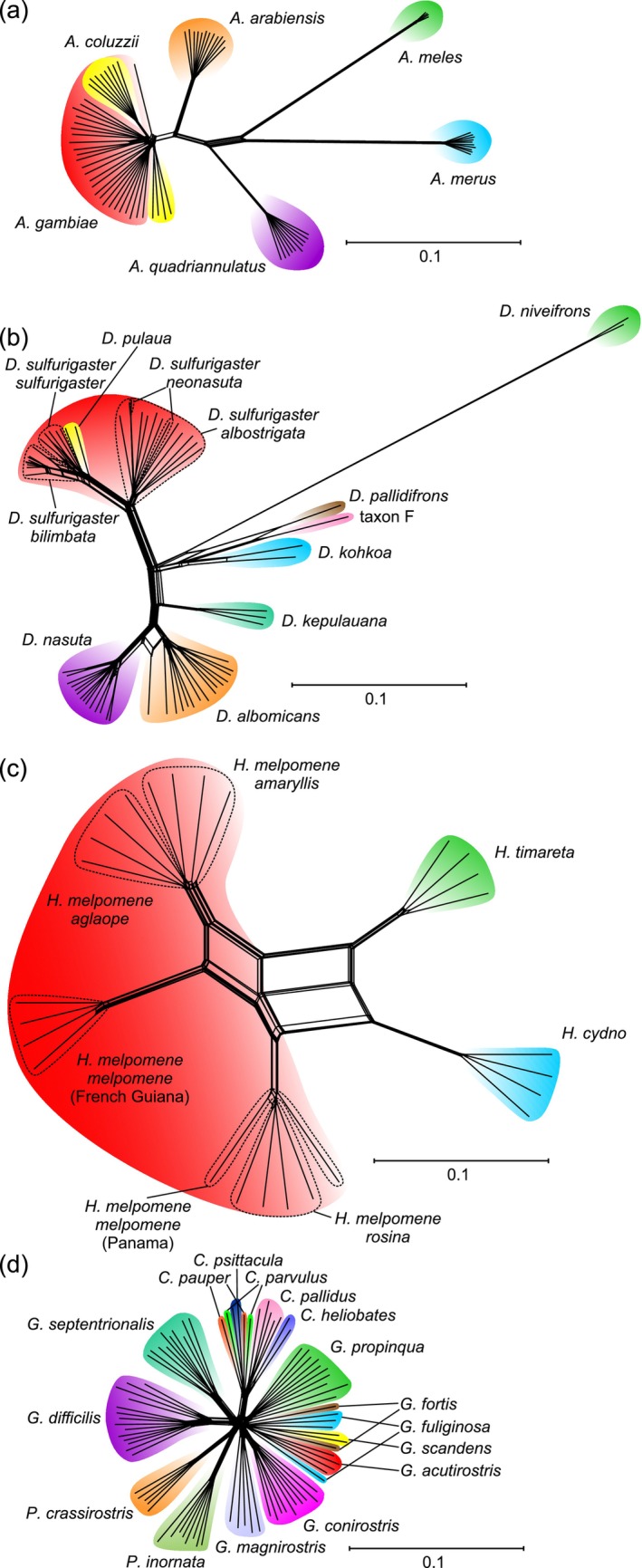
Neighbor‐net networks of the four studied species complexes computed using shared allele distances between SNP data derived from the BUSCO dataset (drawn at the same scale). (a) *Anopheles gambiae* species complex. (b) *Drosophila nasuta* complex. (c) *Heliconius* butterflies. (d) Darwin's finches.

The outcomes of the species delimitation approaches based on the various data assemblies of the four investigated radiations are summarised in Table 1 and Table S1 and illustrated in Figures 1 and Figure S1. The relationships within the radiations are also visualised by Neighbor‐Net networks based on the BUSCO dataset (Figure 2). The Neighbor‐Net networks based on the OrthoDB9 and OrthoDB10 datasets exhibit no qualitative differences. In contrast to the species trees presented in Figure 1 for reference, the networks also indicate the occurrence of reticulations.

**TABLE 1:** Comparison of species classifications. Numbers in parentheses refer to modified classifications suggested in the text.

	Species delimitation approach	Assembly approach	Number of species	Number of individuals	Species delimited	Species delimited in accordance with the current classification	Species delimited in accordance with the current classification (%)	Individuals assigned to the same species as in the current classification	Individuals assigned to the same species as in the current classification (%)
*Anopheles*	tr2	BUSCO	6	74	19	3 (4)	50 (75)	48	65
		OrthoDB9	6	74	11	3 (4)	50 (75)	52	70
		OrthoDB10	6	74	13	3 (4)	50 (75)	62	84
	soda	BUSCO	6	74	17	2 (3)	33 (50)	47	64
		OrthoDB9	6	74	27	1 (2)	17 (33)	38	51
		OrthoDB10	6	74	26	1 (2)	17 (33)	41	55
	structure	BUSCO	6	74	6	4 (6)	67 (100)	70 (74)	95 (100)
		OrthoDB9	6	74	6	4 (6)	67 (100)	70 (74)	95 (100)
		OrthoDB10	6	74	6	4 (6)	67 (100)	70 (74)	95 (100)
*Drosophila*	tr2	BUSCO	9 (8)	67	29	3 (4)	33 (50)	26 (29)	30 (43)
		OrthoDB9	9 (8)	67	34	2 (3)	22 (38)	21 (23)	24 (34)
		OrthoDB10	9 (8)	67	31	3 (4)	33 (50)	24 (27)	28 (40)
	soda	BUSCO	9 (8)	67	39	2 (3)	22 (38)	18 (20)	21 (30)
		OrthoDB9	9 (8)	67	42	2 (3)	22 (38)	17 (19)	20 (28)
		OrthoDB10	9 (8)	67	40	2 (3)	22 (38)	17 (19)	20 (28)
	structure	BUSCO	9 (8)	67	8	5 (8)	56 (100)	52 (67)	60 (100)
		OrthoDB9	9 (8)	67	8	5 (8)	56 (100)	52 (67)	60 (100)
		OrthoDB10	9 (8)	67	8	5 (8)	56 (100)	52 (67)	60 (100)
*Heliconius*	tr2	BUSCO	3	26	6	2	67	16	62
		OrthoDB9	3	26	8	2	67	15	58
		OrthoDB10	3	26	8	2	67	15	58
	soda	BUSCO	3	26	7	2	67	16	62
		OrthoDB9	3	26	7	2	67	16	62
		OrthoDB10	3	26	7	2	67	16	62
	structure	BUSCO	3	26	5	2	67	16	62
		OrthoDB9	3	26	5	2	67	16	62
		OrthoDB10	3	26	5	2	67	16	62
Darwin's finches	tr2	BUSCO	16	87	6	0	07	15	17
		OrthoDB9	16	87	1	0	0	14	16
		OrthoDB10	16	87	1	0	0	14	16
	soda	BUSCO	16	87	12	1	6	38	44
		OrthoDB9	16	87	7	3	19	41	47
		OrthoDB10	16	87	7	2	13	32	37
	structure	BUSCO	16	87	10	8	50	75	86
		OrthoDB9	16	87	12	6	38	73	84
		OrthoDB10	16	87	11	6	38	73	84
All radiations, averaged across datasets	tr2		34 (33)	254	55.67	7.67 (9.67)	23 (29)	107.33 (110.00)	42 (43)
	soda		34 (33)	254	79.33	7.33 (9.33)	22 (28)	112.33 (114.33)	44 (45)
	structure		34 (33)	254	30.00	17.67 (22.67)	52 (69)	211.67 (230.67)	83 (91)

The dataset of the *Anopheles gambiae* complex (Figure 2a) comprises 74 individuals, which have been classified into six species by Fontaine et al. (2015). tr2 classified the individuals into 11–19 clusters, contingent on the data assembly, whereas soda classified them into 17–27 clusters (Figure 1). The structure analyses favoured six clusters across all three datasets. The species delimitation results obtained with tr2 were found to be in agreement with the classification of *A. arabiensis*, *A. merus* and *A. quadriannulatus*, while *A. coluzzii*, *A. gambiae* and *A. melas* were each split into multiple clusters. With the BUSCO dataset, soda delimited *A. merus* and *A. quadriannulatus* according to the current classification, while with the other two datasets only *A. quadriannulatus* was delimited according to the current classification, and all other species were split into several clusters. The structure‐based analyses identified all six species but assigned four individuals from Burkina Faso, previously classified as *A. coluzzii* by Fontaine et al. (2015), to *A. gambiae* . The results indicated that these four individuals exhibited a high degree of admixture, with a greater proportion of their genome derived from *A. gambiae* than from *A. coluzzii* (Figure 1). Also, all individuals classified as *A. gambiae* have a notable proportion of their genome derived from *A. coluzzii*. It may therefore be proposed that the four disputed individuals be more appropriately classified as *A. gambiae* or as hybrids, with no assignment to any species. The four individuals were classified as one or two distinct species by tr2 and soda, respectively. In the sensitivity analyses of the structure‐based approach, *A. gambiae* and *A. coluzzii* were merged with *K* = 7, whereas the best solution calculated with *K* = 9 matched with that calculated with *K* = 8 (Figure S1, Table S1). Due to the merging of *A. gambiae* and *A. coluzzii* in the structure run with *K* = 7, the percentage of individuals classified in accordance with the current classification was reduced to 84%.

tr2 assigned 64%–84% of the *Anopheles* individuals to the same species as in the current classification. soda assigned only 51%–64% of the individuals to the same species as in the current classification, whereas 95% of the individuals were assigned to the same species as in the current classification in the classification derived from the structure results. If the four disputed individuals are classified as *A. gambiae* , all species and all individuals are classified in accordance with the current classification in the classification derived from the structure results.

The Neighbor‐Net network (Figure 2a) demonstrates that all species except *A. coluzzii* and *A. gambiae* are distinctly separated. *Anopheles gambiae* represents a bush‐like radiation, with the (almost) pure *A. coluzzii* separated by a distinct branch and the strongly admixed *A. coluzzii* forming part of the bush. *Anopheles gambiae* is paraphyletic in the species tree (Figure 1).

The *Drosophila nasuta* species complex (Figure 2b) dataset comprises 67 individuals, representing nine species according to the classification presented by Mai et al. (2019). tr2 classified the individuals into 29–34 clusters, soda into 39–42 clusters and structure into 8 clusters. Only the poorly represented species *D. kohkoa*, *D. kepulauana* and *D. niveifrons* (with 2–3 individuals each) were delimited with tr2 based on the BUSCO and OrthoDB10 datasets in accordance with the current classification. It should be noted that the sample designated ‘d.niveifrons_O‐30’ was classified as *D. pulaua* by Mai et al. (2019; table S1) and in the GenBank entry. Only *D. kohkoa* and *D. niveifrons* were delimited by tr2 based on the OrthoDB9 dataset and by soda in accordance with the current classification. No approach separated *D. pallidifrons* and ‘taxon F', which were represented by a single individual each. The structure analyses delimited *D. albomicans*, *D. kohkoa*, *D. kepulauana*, *D. nasuta* and *D. niveifrons* in accordance with the current classification. However, the *D. sulfurigaster* complex was split into two clusters, one including *D. sulfurigaster albostrigata* and *D. sulfurigaster neonasuta*, and the other including *D. s. sulfurigaster*, *D. sulfurigaster bilimbata* and *D. pulaua*. The species classifications derived from the structure runs based on the BUSCO dataset with *K* = 8 and *K* = 10 matched with that derived from the run with the highest likelihood with *K* = 9 (Figure S1, Table S1). Only 24%–30% of the individuals were assigned to the same species as in the current classification by tr2 and 20%–21% by soda. The degree of congruence between the structure results and the classification presented by Mai et al. (2019) is similarly low, with 60% of the individuals assigned to the same species as in the classification of Mai et al. (2019). The primary source of incongruence is the splitting of the *D. sulfurigaster* complex into two distinct species, a classification that may be substantiated (see Species validation below). If the division of the *D. sulfurigaster* complex into two species and the synonymy of ‘taxon F' with *D. pallidifrons* is accepted, all species and all individuals were correctly classified in the classifications derived from the structure results with the highest likelihoods.

The Neighbor‐Net network of the *Drosophila nasuta* species complex (Figure 2b) shows the same reticulation between *D. kohkoa*, *D. pallidifrons* and ‘taxon F'. A single hybrid specimen exists between the otherwise clearly separated *D. albomicans* and *D. nasuta* (with 61% ancestry from *D. albomicans* and 37% from *D. nasuta* , as determined by the structure analysis of the BUSCO dataset). In accordance with the findings of the structure analyses, the *D. sulfurigaster* complex is clearly divided into two clusters in the Neighbor‐Net, comprising one cluster that encompasses *D. sulfurigaster albostrigata* and *D. sulfurigaster neonasuta*, and another cluster that includes *D. s. sulfurigaster*, *D. sulfurigaster bilimbata* and *D. pulaua*.

The *Heliconius* dataset (Figure 2c) comprises 26 individuals, classified by Martin et al. (2013) into three species. tr2 classified the individuals into 6–8 clusters, soda into 7 clusters and structure into 5 clusters. The species classifications derived from the structure runs based on the BUSCO dataset with *K* = 9 and *K* = 11 matched with that derived from the run with the highest likelihood with *K* = 10 (Figure S1, Table S1). All approaches delimited *H. cydno* and *H. timareta* in accordance with the current classification, each of which is represented by four individuals from a single region. In contrast, all approaches subdivided *H. melpomene*, which is represented by several subspecies from different regions into several clusters. The clusters exhibited partial correspondence with subspecies. However, no approach grouped *H. m. melpomene* from French Guiana with the individuals from Panama, which were identified as *H. m. melpomene* due to their colour pattern, despite the fact that they are genetically closer to the populations classified as *H. melpomene rosina* from Panama. The proportion of individuals assigned to the same species as in the current classification by tr2 was 58%–62%, while soda and structure achieved 62%. The structure analyses revealed evidence of admixture between *H. timareta* from Peru and the Peruvian samples of *H. melpomene* (2.2%–5.1% in *H. timareta* and 0%–3.0% in *H. melpomene*) and between *H. cydno* from Panama and the samples of *H. melpomene* from Panama (0% in *H. cydno* and 0%–2.8% in *H. melpomene*).

The Neighbor‐Net network (Figure 2c) showed that the geographically widely separated populations of *H. melpomene* exhibited a similar degree of distinctiveness from each other as they did from *H. cydno* and *H. timareta*. It confirmed the reticulation between *H. timareta* from Peru and the Peruvian samples of *H. melpomene*, as well as between *H. cydno* and the samples of *H. melpomene* from Panama. Furthermore, the samples from Panama identified as ‘*H. m. melpomene*’ were grouped with *H. melpomene rosina* from Panama and not with *H. m. melpomene* from French Guiana.

The Darwin's finches dataset (Figure 2d) comprises 87 individuals belonging to 16 species, as classified by Lamichhaney et al. (2015). tr2 classified the individuals into six clusters based on the BUSCO dataset. In contrast, tr2 grouped all individuals into a single cluster based on the two other datasets. soda suggested a classification into 12 clusters based on the BUSCO dataset, while seven clusters are delimited based on the two other datasets. Based on the structure results, the individuals were classified into 10–12 clusters. No species was delimited in accordance with the current classification with tr2. soda delimited only 1–3 species in accordance with the current classification, whereas 6–8 species were classified in accordance with the current classification based on the structure analyses with *K* = 13. The proportion of individuals assigned to the same species as in the current classification by tr2 was 16%–17% and 37%–47% by soda. 84%–86% of the individuals were assigned to the same species as in the current classification in the structure‐based analyses. The number of species delimited in accordance with the current classification dropped to 7 in the classification based on the structure analyses based on the BUSCO dataset with *K* = 12 and *K* = 14 (Figure S1, Table S1). 82% and 84% of the individuals, respectively, were assigned to the same species as in the current classification in these analyses.

The Neighbor‐Net network of Darwin's finches (Figure 2d) shows that this is the most star‐like of the studied radiations, exhibiting the shortest distances among the species. Several of the undersampled *Camarhynchus* and *Geospiza* species are not well resolved in the network.

### 3.2 Species Validation by Isolation‐By‐Distance Tests

The primary species hypotheses, for which a sufficient number of samples were available, were tested using IBD tests with all datasets. Only the results based on the BUSCO dataset are shown because the IBD tests based on the OrthoDB9 and OrthoDB10 datasets yielded results that were qualitatively similar to those based on the BUSCO dataset.

**FIGURE 3:**
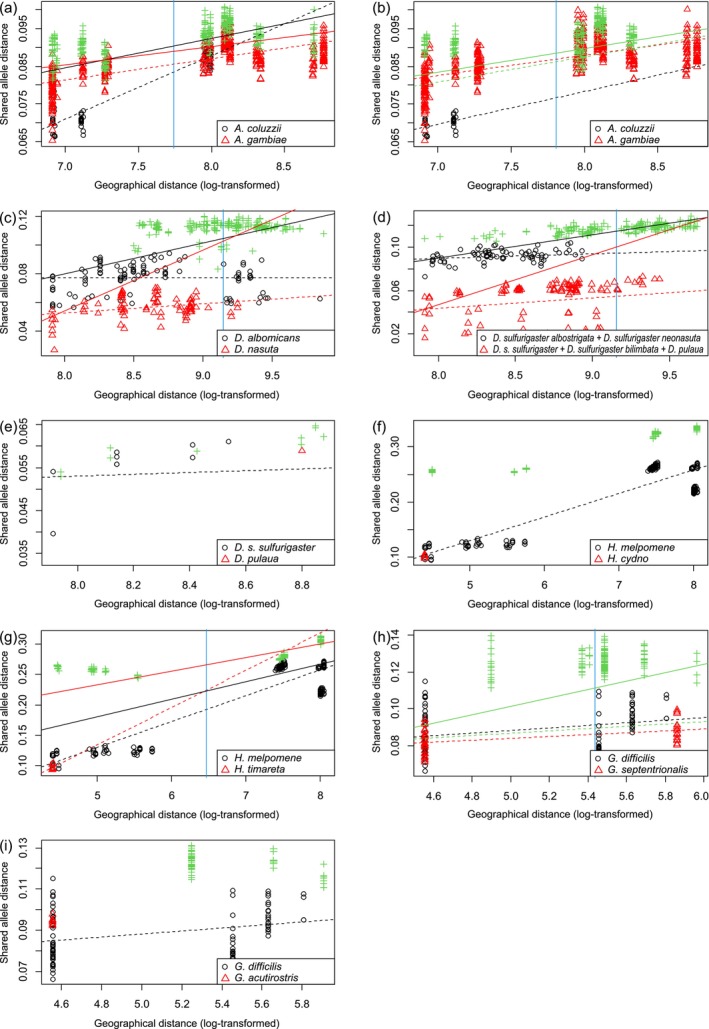
Relationships between genetic and log geographical distances in pairs of individuals of two primary species hypotheses. Black circles and red triangles indicate distances between individuals belonging to the first and second primary species hypothesis, respectively. Green crosses indicate distances between individuals belonging to different primary species hypotheses. Black and red dashed lines are regression lines fitted within the first and second primary species hypothesis, respectively. Blue lines indicate centres of the between‐group geographical distances. In cases where the relationship between genetic and geographical distances within the two primary species hypotheses can be modelled by a single regression (*H*_01_ not rejected), the green dashed line is the regression line fitted to the withingroup distances only (i.e. the black circles and red triangles taken together), and the green solid line is the regression line fitted to all distances together. When the relationship between genetic and geographical distances within the two primary species hypotheses cannot be modelled by a single regression (*H*_01_ rejected), the black and red solid lines are regression lines fitted to the distances within the first and second primary species hypothesis, respectively, together with the distances between the two primary species hypotheses. See Table 2 for details.

IBD tests confirmed that the differentiation between *Anopheles gambiae* and *A. coluzzii* cannot be attributed to isolation by distance (Table 2) despite the admixture between these two taxa (Figure 1). This result is not contingent on whether the four disputed specimens from Burkina Faso are classified as *A. coluzzii* (Table 2, Figure 3a), as proposed by Fontaine et al. (2015), or as *A. gambiae* (Table 2, Figure 3b), given their higher ancestry proportion from *A. gambiae* .

**TABLE 2:** IBD tests of the primary species hypotheses based on the BUSCO dataset.

Primary species hypotheses	*H*_01_	*H*_02_	*H*_03_	Figure 3
*Anopheles coluzzii* vs. *A. gambiae* classified according to Fontaine et al. (2015)	< 10^−3^		< 10^−6^/< 10^−6^	a
*A. coluzzii* vs. *A. gambiae* classified according to the structure results	0.129	0.004		b
*D. albomicans* vs. *D. nasuta*	< 10^−4^		< 10^−7^/< 10^−12^	c
*D. sulfurigaster albostrigata* + *D. sulfurigaster neonasuta* versus *D. s. sulfurigaster* + *D. sulfurigaster bilimbata* + *D. pulaua*	< 10^−5^		0.001/< 10^−5^	d
*D. pulaua* vs. *D. s. sulfurigaster*	—		−/0.147	e
*H. cydno* vs. *H. melpomene*	—		−/< 10^−5^	f
*H. timareta* vs. *H. melpomene*	—		−/< 10^−4^	g
*G. difficilis* vs. *G. septentrionalis*	0.706	< 10^−5^		h
*G. acutirostris* vs. *G. difficilis*	—		−/0.010	i

*Note: p*‐values were obtained for the hypothesis that the regressions of genetic on geographical distances within two primary species hypotheses agree (*H*_01_). If this hypothesis could not be rejected, the hypothesis that the regression pattern between primary species hypotheses is compatible with the regression based on the combined within‐group data was tested (*H*_02_). If *H*_01_ was rejected, the hypothesis that the regression pattern between groups is compatible with the regression within the primary species hypotheses was tested for each of the primary species hypotheses separately (*H*_03_).

IBD tests also corroborated the classification of *Drosophila albomicans* and *D. nasuta* as distinct species (Table 2, Figure 3c). IBD tests demonstrated that the divergent population groups *D. sulfurigaster albostrigata*, including *D. sulfurigaster neonasuta*, versus *D. s. sulfurigaster*, *D. sulfurigaster bilimbata* and *D. pulaua* (Figures 1 and 2b), which are considered two primary species hypotheses based on the structure analysis, exhibited greater differentiation than would be expected based on their geographical distance (Table 2, Figure 3d), providing support for their classification as distinct species. Because *D. pulaua* represents the sister group of *D. s. sulfurigaster* in the tree based on markers from the X chromosome (Mai et al. 2019; fig. 4) and Mai et al. (2019) suggested that this tree reflects the true species branching order, we tested whether the differentiation between *D. pulaua* and *D. s. sulfurigaster* can be explained by IBD. This hypothesis cannot be rejected (Table 2, Figure 3e), indicating that *D. pulaua* is conspecific with *D. sulfurigaster*.

Notwithstanding the admixture between *H. cydno* and *H. timareta* and the regional *H. melpomene* populations, IBD tests substantiated the classification of *H. cydno* and *H. timareta* as distinct species (Table 2, Figure 3f).

IBD tests showed that the populations of Darwin's finches previously classified as *Geospiza difficilis* from Wolf and Darwin (now *G. septentrionalis* ) and from Genovesa (now *G. acutirostris* ) are genetically more strongly differentiated from the populations from Pinta, Fernandina and Santiago ( *G. difficilis* in the strict sense) than expected based on isolation by distance (Table 2, Figure 3h,i).

## 4 Discussion

### 4.1 Species Discovery by Approaches Based on the MSC Model versus Population Genetic Methods: Theoretical Assumptions and Practical Consequences

The two approaches to species delimitation, which are compared here, methods based on the MSC model and a two‐step approach combining population genetic methods with IBD tests, make different assumptions about the nature of species. It is therefore not surprising that the results also differ. The MSC model assumes that the alleles of a species coalesce completely at random, i.e. there is no structure within species, and there is no gene flow after species divergence, i.e. a sudden transition from panmixis to complete separation (Fujisawa et al. 2016; Rabiee and Mirarab 2020; Rannala and Yang 2003). In contrast, the two‐step approach combining population genetic methods with IBD tests assumes that species consist of populations separated by extrinsic barriers and that there is a dispersal limitation that results in increasing genetic distances with increasing geographic distances, i.e. isolation by distance (Wright 1943).

As can be expected based on the assumption that there is no structure within species, i.e. that each genetically distinct population represents a distinct species, the two approaches based on the MSC model generally resulted in a high number of species (Figure 1). There is a high degree of over‐splitting relative to the current classifications across all four studied species complexes, with tr2 delimiting 164% of the species number originally identified and soda reaching 233%. Furthermore, the proportion of species delimited according to the current classification was low, at 23% for tr2 and 22% for soda, while the proportion of individuals assigned to the same species as in the current classification was similarly low, at 42% for tr2 and 44% for soda (Table 2). The proportions of species delimited according to the current classification and of individuals assigned to the same species as in the current classification only marginally increase if the proposed taxonomic changes (classifying the *A. coluzzii*/*gambiae* hybrids with a higher ancestry proportion of the genome from *A. gambiae* than from *A. coluzzii* as *A. gambiae* ; classifying *D. albostrigata* (including *D. s. neonasuta*) and *D. sulfurigaster* (including *D. s. pulaua* as a subspecies) as distinct species; and synonymizing ‘taxon F' with *D. pallidifrons*) are accepted. Similar over‐splitting by methods based on the MSC model has been reported in several studies (e.g., Bamberger et al. 2022; Barley et al. 2018; Chambers and Hillis 2020; Dietz et al. 2024; Leaché et al. 2019; Sukumaran and Knowles 2017). The fact that the results of approaches based on the MSC model often result in an over‐splitting compared to current classifications indicates that many systematic biologists do not accept the assumption of the MSC model that populations that are not panmictic represent distinct species.

The other assumption of the MSC model, that there is no gene flow after species divergence (Fujisawa et al. 2016; Rabiee and Mirarab 2020; Rannala and Yang 2003), has less predictable consequences. It has probably contributed to the lumping of the species of Darwin's finches by tr2 and soda (Figure 1), whereas in the other groups admixed individuals or groups of individuals were even misclassified as distinct species by these programs, e.g. the admixed *A. gambiae* /*coluzzii* specimens from Burkina Faso, admixed specimens of *D. sulfurigaster* (including *D. pulaua*), or an admixed *D. albomicans*/*nasuta* specimens (Figure 1).

The primary objective of structure is also to identify population structure, but unlike the MSC model, it allows admixture between populations (Pritchard et al. 2000). In comparison to the MSC model‐based approaches, structure tends to favour larger clusters, which are typically in better agreement with existing classifications (Table 2). On average, there is even a slight underestimation, with 88% of the original species numbers, which is primarily due to the lumping of species of Darwin's finches (Table 2). Consequently, the proportion of species delimited according to the current classification is more than twice as high as that of the MSC‐based approaches, although 52% remains unsatisfactory. However, this figure would rise to 69% if the aforementioned taxonomic alterations were to be accepted. Furthermore, the percentage of individuals assigned to the same species as in the current classification is 83%, which is almost twice as high as that of the MSC‐based approaches, and would also increase to 91% if the aforementioned taxonomic changes were to be accepted (Table 2).

One problem with population genetic approaches such as structure is the identification of an appropriate number of ancestral populations (*K*). There are different statistics for determining the most appropriate *K* (Alexander et al. 2009; Evanno et al. 2005; Pritchard et al. 2000), but there is no consensus on the most effective approach. Therefore, I examined the changes in the proposed species delimitation when the *K* found in the run with the highest likelihood is decreased or increased by 1 (Figure S1, Table S1). In the *Drosophila nasuta* and *Heliconius melpomene* complexes, a variation of *K* by 1 had no influence on the resulting species combination because ‘ghost clusters’ which did not represent the largest proportion of ancestry in any of the examined individuals and might represent populations that have not been sampled, that are no longer extant, or that are computational artefacts, were either merged or separated. In the *Anopheles gambiae* complex, an increase in *K* by 1 had also no effect, whereas a decrease resulted in the merging of *A. gambiae* and *A. coluzzii*, which are connected by admixture. In Darwin's finches, variations of *K* by minus and plus 1 resulted in different assignments of individuals of underrepresented, closely related species. Consequently, the fuzziness of the correct number of ancestral populations has a minor influence on the results given an appropriate sampling. It is meaningful to explore the consequences of varying *K* within a reasonable range. An over‐splitting of a species by a too high *K* may be corrected in the species validation step. If IBD tests show that the differentiation between primary species hypotheses recognised only with higher *K* can be explained by isolation by distance, they can be merged again into secondary species hypotheses so that the secondary species hypotheses are less sensitive to overestimates of *K* as long as a sufficient number of populations of each potential unit is sampled. Some approaches for identifying an appropriate *K* are biased towards *K* = 2, even when more populations are present (Janes et al. 2017). If *K* = 2 is found, potential structure within the two identified clusters should be examined by re‐running structure on the two populations separately.

structure (Pritchard et al. 2000) and similar population genetic approaches, such as admixture (Alexander et al. 2009), were developed with the objective of delimiting populations in a manner that optimises the attainment of Hardy–Weinberg equilibrium within each population and to estimate individual ancestry and admixture. The objective is to optimise a criterion based on population genetic theory. Therefore, the downgrading of these methods as “phenetic” (Dietz et al. 2024) is inappropriate. It is acknowledged that these methods do not take into account the information provided by gene trees. However, the evolution within species is dominated by changes in allele frequencies and gene flow, which are not considered by species delimitation methods based on the MSC model. In order to gain a comprehensive understanding of the natural history of a group of species, it is necessary to combine population genetic approaches, which are capable of delimiting populations and detecting admixture, with phylogenetic approaches, which are able to reconstruct the relationships between units based on the information in gene trees. The identification of admixture between individuals and populations is an important indication that species status should be further tested, but it is not a proof that taxa are not species. Species status depends on the level and distribution of admixture. The IBD test can be used to decide whether the observed level of admixture between primary species hypotheses is compatible with the hypothesis that these are distinct species or whether they should be merged.

structure and similar approaches were not designed to identify species boundaries. The nature of the clusters that are delimited is contingent upon the sampling. If populations of a geographically differentiated species are subjected to intensive sampling, the program will delimit populations or population groups (in the event of pronounced differentiation, these may be classified as subspecies). If several species, but few individuals per species are sampled, the optimisation of genetically homogeneous clusters by the algorithm will result in the delimitation of species.

If geographically widely separated populations of a species are sampled, there is a risk of over‐splitting, whereby conspecific populations or population groups are classified as separate species, as exemplified by *Heliconius melpomene*. In the case of this taxon, populations from Panama, French Guiana and Peru were sampled, but not from intermediate areas of the continuous range. In the absence of information about geographical intermediate populations, it is challenging to determine whether the splitting of a widespread taxon into multiple species is justified. As demonstrated by Martin et al. (2016), the samples identified as ‘*H. m. melpomene*’ from Panama form a distinct group with the samples of *H. melpomene rosina* from Panama, *H. melpomene vulcanus* from Panama and western Colombia, and *H. melpomene cythera* from western Ecuador. The samples from French Guiana identified as ‘*H. m. melpomene*’ constitute a second group, which also includes populations from the eastern slope of the Andes and from Brazil (Van Belleghem et al. 2018). The samples identified as ‘*H. m. melpomene*’ from the eastern slope of the Andes in northern Colombia belong to the first group but show admixture with the second group. The populations from Panama and northern Colombia identified as ‘*H. m. melpomene*’ cannot be assigned to the same subspecies as those from French Guiana even if they have a similar wing pattern. A revision of the subspecies classification of these populations is therefore necessary.

A more significant issue than the over‐splitting of well‐represented groups is the undersampling of rare or geographically restricted taxa (Lim et al. 2012). Approaches based on the maximisation of Hardy–Weinberg equilibrium within populations tend to result in the merging of such underrepresented groups (Bamberger et al. 2022; Kalinowski 2011; Puechmaille 2016). This is because the gain with regard to Hardy–Weinberg equilibrium may be larger when a large, slightly heterogeneous group is split into two homogeneous clusters than when a small, strongly heterogeneous cluster is split into two still heterogeneous clusters. The polyphyly of their components in a species tree and the apparent descent from multiple ancestral populations may be used to identify heterogeneous clusters in structure results. Potentially heterogeneous clusters may be assessed by increasing the number of representatives sampled from the various components of these artificial clusters.

The issue of undersampling is particularly evident in the study of Darwin's finches. The dataset presented by Lamichhaney et al. (2015) included representatives of only a single island for each of the five nominal species of *Camarhynchus*, the tree finches. However, three of the five species are known to occur on several islands (Zink and Vázquez‐Miranda 2019), and four of the five species were represented by only two specimens. Given the limitations of the sampling strategy employed, it is challenging to delimit species correctly (what was not the primary objective of the original study). All species discovery approaches resulted in the merging of the five species. To test the hypothesis that these nominal species are in fact coherent natural clusters and not similar morphotypes that originated by convergent selection on different islands, it is necessary to obtain genomic data from several islands (or different parts of larger islands) (see also Zink and Vázquez‐Miranda 2019). The same is true for the complex of the *Geospiza* species *G. fortis* , *G. fuliginosa* and *G. scandens* , which were also merged by all species discovery approaches.

The *Drosophila nasuta* complex (Figure 1) provides an example in which the approach based on structure revealed new primary species hypotheses that were not discussed by Mai et al. (2019) or Dietz et al. (2024). However, their phylogenetic analyses already indicated a deep split between *D. sulfurigaster albostrigata* and *D. sulfurigaster neonasuta* on the one side, and *D. s. sulfurigaster*, *D. sulfurigaster bilimbata* and *D. pulaua* on the other. tr2 split the *D. sulfurigaster* complex (including the two specimens labelled *D. pulaua*) into 15–18 primary species hypotheses, and soda into 18–20 primary species hypotheses. In contrast, structure recognised two clusters, corresponding to the two aforementioned groups in the tree.

The two individuals from India classified as *D. sulfurigaster neonasuta* by Mai et al. (2019) do not form a monophyletic group (Figure 1). The specimen from Mysore is sister to a specimen from Cambodia that has been classified as *D. sulfurigaster albostrigata*, whereas the specimen from Coimbatore is sister to a specimen from Sri Lanka that has also been classified as *D. sulfurigaster albostrigata*. The latter two specimens are related to a group of specimens from Cambodia and Thailand. This suggests that *D. sulfurigaster albostrigata* has colonised the Indian Peninsula on at least two occasions, originating from Southeast Asia. Furthermore, it lends support to the hypothesis that *D. sulfurigaster neonasuta* is a synonym of *D. sulfurigaster albostrigata*, as suggested previously by Mai et al. (2019).

Another case where a change to the classification presented by Mai et al. (2019) may be appropriate concerns *D. pallidifrons* and ‘taxon F'. Both taxa are only represented by a single individual in the dataset. They are classified by the MSC‐based approaches as well as by the approach based on structure as a single species (Figure 1). The two individuals are sister groups in the tree and the split separating them is not deeper than between individuals of other species. Neither Mai et al. (2019) nor Kitagawa et al. (1982), who introduced ‘taxon F', justified its distinctness. Obviously, more specimens should be examined of both taxa, but as long as no arguments for their distinctness are known, it seems more appropriate to consider ‘taxon F' conspecific with *D. pallidifrons*.

Finally, the identity of the *Anopheles* specimens from Burkina Faso that Fontaine et al. (2015) assigned to *A. coluzzii* is controversial. These four individuals exhibited a high degree of admixture, with a greater proportion of their genome derived from *A. gambiae* than from *A. coluzzii* (Figure 1). Nothing is known about other characters that could justify their classification as *A. coluzzii*. Fontaine et al. (2015) argued that the dominant tree topologies of the autosomes and the X chromosome in the *A. gambiae* complex differ due to autosomal introgression. Therefore, the clustering of the *Anopheles* specimens from Burkina Faso with *A. gambiae* in the tree based on markers on the X chromosome (Fontaine et al. 2015; figure S20) is further evidence for their closer relationship to *A. gambiae* than to *A. coluzzii*. These hybrids should not be classified as *A. coluzzii*. It is a question of the amount of admixture, especially with regard to species specific alleles, whether hybrids should be classified in one of the parental species.

The species classifications obtained based on genomic datasets of four radiations were consistent across the three USCO datasets assembled using disparate strategies, with the exception of a few undersampled Darwin's finches (Figure 1, Table 1). The precise composition of the datasets is not decisive for species delimitation, provided that the datasets contain a substantial number of unlinked markers that are neutral or subject to similar selection pressures across all species.

### 4.2 Integrative Taxonomy: Power of Geographical Data

Neither approaches based on the MSC model initially designed for species delimitation (Fujita et al. 2012; Knowles and Carstens 2007; Yang and Rannala 2014) nor population genetic approaches initially designed for delimiting populations within species (Alexander et al. 2009; Pritchard et al. 2000) incorporate criteria that enable the differentiation between clusters corresponding to species and clusters representing other differentiation levels (Sukumaran et al. 2021). The results of the present study, as well as those of previous research, demonstrate that all these approaches may identify clusters of individuals representing diverse levels of the biological hierarchy, from local demes to differentiated populations connected by gene flow, up to species without gene flow and higher taxa (Bamberger et al. 2022; Barley et al. 2018; Chambers and Hillis 2020; Dietz et al. 2024; Leaché et al. 2019; Sukumaran and Knowles 2017). Therefore, integrative approaches that use other types of data to further test the primary species hypotheses based on genomic data are crucial (Edwards and Knowles 2014; Guillot et al. 2012; Padial et al. 2010; Rittmeyer and Austin 2012; Sauer and Hausdorf 2012; Solís‐Lemus et al. 2015).

The application of integrative taxonomy is frequently constrained by the incomplete nature of the morphological or ecological datasets pertaining to the individuals under investigation. The collection of such data is often challenging, resulting in matrices that are frequently incomplete. In contrast, geographical data are typically accessible with relative ease. In order to test the species status of primary species hypotheses using geographic data, it is necessary to have a model of the variation of genetic data with geographic data within species. The simplest model that describes the relationships between genetic and geographical distances within species is the isolation by distance model, which was first proposed by Wright (1943). The IBD test, as outlined by Hausdorf and Hennig (2020), can be used to test whether the differentiation between two primary species hypotheses can be explained by the relationship between genetic and geographical distances within the clusters. IBD tests represent a powerful tool for investigating whether it is justifiable to classify two groups of allopatric populations as distinct species or whether the differentiation between the population groups can be explained by IBD.

The IBD tests (Table 2) provided evidence in support of the classification of *Anopheles coluzzii* as a distinct species (Fontaine et al. 2015). Furthermore, the results supported the classification of *Drosophila albomicans* and *D. nasuta* as separate species by Kitagawa et al. (1982) and Mai et al. (2019), despite the absence of pre‐mating isolation. This stands in contrast to the opinion of Ponnanna et al. (2021), who classified these taxa as subspecies. The IBD tests also supported the separation of the populations of Darwin's finches previously classified as *Geospiza difficilis* from Wolf and Darwin (now *G. septentrionalis* ) and from Genovesa (now *G. acutirostris* ) as distinct species from the populations from Pinta, Fernandina and Santiago ( *G. difficilis* in the strict sense), as proposed by Lamichhaney et al. (2015) (Table 2). Furthermore, an IBD test corroborated that the two population groups of the *D. sulfurigaster* complex identified by structure (Figure 1) exhibit a greater degree of differentiation than anticipated based on the correlation between genetic and geographical distances within these groups (Table 2, Figure 3d). It is therefore proposed that *D. sulfurigaster* and *D. albostrigata* (including *D. s. neonasuta* as a synonym) be classified as separate species. In contrast, an IBD test did not support the separation of *D. pulaua* from *D. sulfurigaster* as a distinct species (Table 2, Figure 3e). The male courtship song of *D. pulaua* is markedly distinct from that of *D. sulfurigaster* (Mai et al. 2019), yet the two taxa exhibit only partial reproductive isolation. Females of *D. sulfurigaster* have been observed to produce fertile F_1_ progeny in crosses with *D. pulaua*, whereas no offspring have been obtained from matings of *D. pulaua* females with *D. sulfurigaster* males (Kitagawa et al. 1982). It is important to note that IBD tests should not be considered the definitive proof of species status. In the event that gene flow between *D. pulaua* and *D. sulfurigaster* has recently ceased as a result of, for instance, alterations to the courtship song or chromosomal mutations, a reassessment of its status will be required. In the absence of such evidence, however, it seems reasonable to propose that *D. pulaua* should be classified as a subspecies of *D. sulfurigaster*. This is based on the position of *D. pulaua* within *D. sulfurigaster* in the species tree (Figure 1), the lack of recognition of a distinct *D. pulaua* cluster in the structure analysis (Figure 1) and the IBD test results (Table 2, Figure 3e).

The case of *Heliconius melpomene*, where only a few populations from Panama, French Guiana and Peru were sampled, and the populations from each region were separated as distinct species by all methods (Figure 1), shows that insufficient sampling across the range of a species can affect species discovery. If there are fewer than three populations sampled in each region, species validation by IBD tests also becomes unfeasible. Accordingly, the planning of sampling for a taxonomic study represents a crucial step. On the one hand, the entire range of a species should be sampled homogeneously. However, this may result in an overrepresentation of widespread species and might lead to a lumping of regionally restricted and therefore undersampled species by species discovery approaches such as structure (Bamberger et al. 2022; Kalinowski 2011; Puechmaille 2016). In the event that prior knowledge exists regarding potential taxa or morphological diversity, it is imperative to ensure the sampling of multiple individuals representing all potential taxa and morphological varieties. In an optimal scenario, at least three populations distributed across the presumed range of each taxon should be sampled, because this is the minimum necessary for the calculation of the relationship between genetic differentiation and geographical distance within a group and, thus, for species validation with IBD tests. This will not be feasible in every case; for example, in the case of local endemics. The distinctness of a local endemic can still be tested against a widespread species, which is represented by three or more populations in a one‐sided IBD test (considering only the relationship between genetic and geographical distances in the widespread species). However, if local endemic species are sister species, their distinctness cannot be corroborated by an IBD test. Nevertheless, their distinctness may be supported by auxiliary criteria, such as a genetic differentiation that is similar to that observed in related species pairs tested by IBD tests.

IBD tests are employed to ascertain whether there is a correlation between genetic distances and (transformed) geographical distances. The interpretation of higher genetic distances than expected based on the geographical distances as evidence for separate species is contingent upon the assumption that the increased genetic distance reflects intrinsic barriers to gene flow. However, extrinsic barriers such as mountain ranges can also impede gene flow, leading to an increase in genetic distances. This should be taken into account when interpreting the results. It may be possible to consider the effects of extrinsic barriers in IBD tests by using resistance distances (McRae 2006; McRae et al. 2008) that reflect the landscape connectivity rather than straight‐line distance.

While the integrative approach proposed here relies on species validation of primary species hypotheses, Sukumaran et al. (2021) proposed to solve the problem that genetic data alone are insufficient to recognise species boundaries by incorporating previous species identifications of a subset of population lineages into an MSC approach. Whereas the species validation approach tests primary species hypotheses with independent data, the approach proposed by Sukumaran et al. (2021) relies on the accuracy of the prior species hypotheses, which are not evaluated, and on the assumption that the speciation‐completion rate estimated based on the known species classification is consistent across the entire studied group.

### 4.3 Recommendations for Taxonomic Studies

The appropriate method for species delimitation depends on the assumptions made about the nature of species. If it is assumed that there is no structure within species, and there is no gene flow after species divergence, methods based on the MSC model such as tr2 (Fujisawa et al. 2016) or soda (Rabiee and Mirarab 2020) are most appropriate for species delimitation. If it is assumed that species may include partially isolated populations, which are connected by gene flow, and that dispersal limitation may result in a pattern of IBD within species, the two‐step approach combining population genetic programs such as structure (Pritchard et al. 2000) or admixture (Alexander et al. 2009) with species validation of the resulting primary species hypotheses, especially those showing admixture, by using IBD tests (Hausdorf and Hennig 2020), is more appropriate. Primary species hypotheses that are less or equally differentiated than would be expected based on IBD can then be merged into secondary species hypotheses. This two‐step approach resulted in species delimitations that were much more similar to the currently accepted classifications of these four well‐studied radiations than the results of approaches based on the MSC model, which resulted in high species over‐splitting, low percentages of species delimited according to the current classification, and low percentages of individuals assigned to the same species as in the current classification. The results of the case studies also showed the importance of sampling at least three populations from the range of each species to assess whether differences between populations can be explained by geographic variation within the species, or whether they indicate distinct species.

## Author Contributions

The author takes full responsibility for this article.

## Disclosure

This research provides information and recommendations about species delimitation with genomic data for the scientific community.

## Conflicts of Interest

The author declares no conflicts of interest.

## Supporting information


Data S1

## Data Availability

No new sequence data were generated during this study. Data for the four case studies are available at NCBI BioProject (https://www.ncbi.nlm.nih.gov/bioproject) under the following accession numbers: Darwin's finches: PRJNA263122 (Lamichhaney et al. 2015), *Anopheles gambiae* complex: PRJNA67511 (Fontaine et al. 2015), *Drosophila nasuta* species complex: PRJNA554139 (Mai et al. 2019), *Heliconius*: PRJEB1749 (Martin et al. 2013).
