# Supplementary material for: Species Delimitation Using Genomic Data: Options and Limitations
Source: Mol Ecol. 2025 Mar 3;34(8):e17717. doi: 10.1111/mec.17717 (PMC11974488; doi:10.1111/mec.17717)
Supplement: Supplementary file 1 — Data S1 [file MEC-34-e17717-s001.pdf]

MOLECULAR ECOLOGY

Supplemental Information for:

Species delimitation using genomic data: options and limitations

Bernhard Hausdorf

FIGURE S1 Species trees of the four radiations, STRUCTURE results (individual ancestry) calculated with three different *K* based on the BUSCO dataset and corresponding species classifications derived from the STRUCTURE results. Background colour and labels on the tree indicate the species classification in the original reference. Coloured boxes in the species classification columns indicate that inferred clusters are congruent with species in the original classifications and curved lines connect partitions belonging together according to the respective classification but are separated by other partitions (partly modified from Dietz et al., 2024).

MOLECULAR ECOLOGY

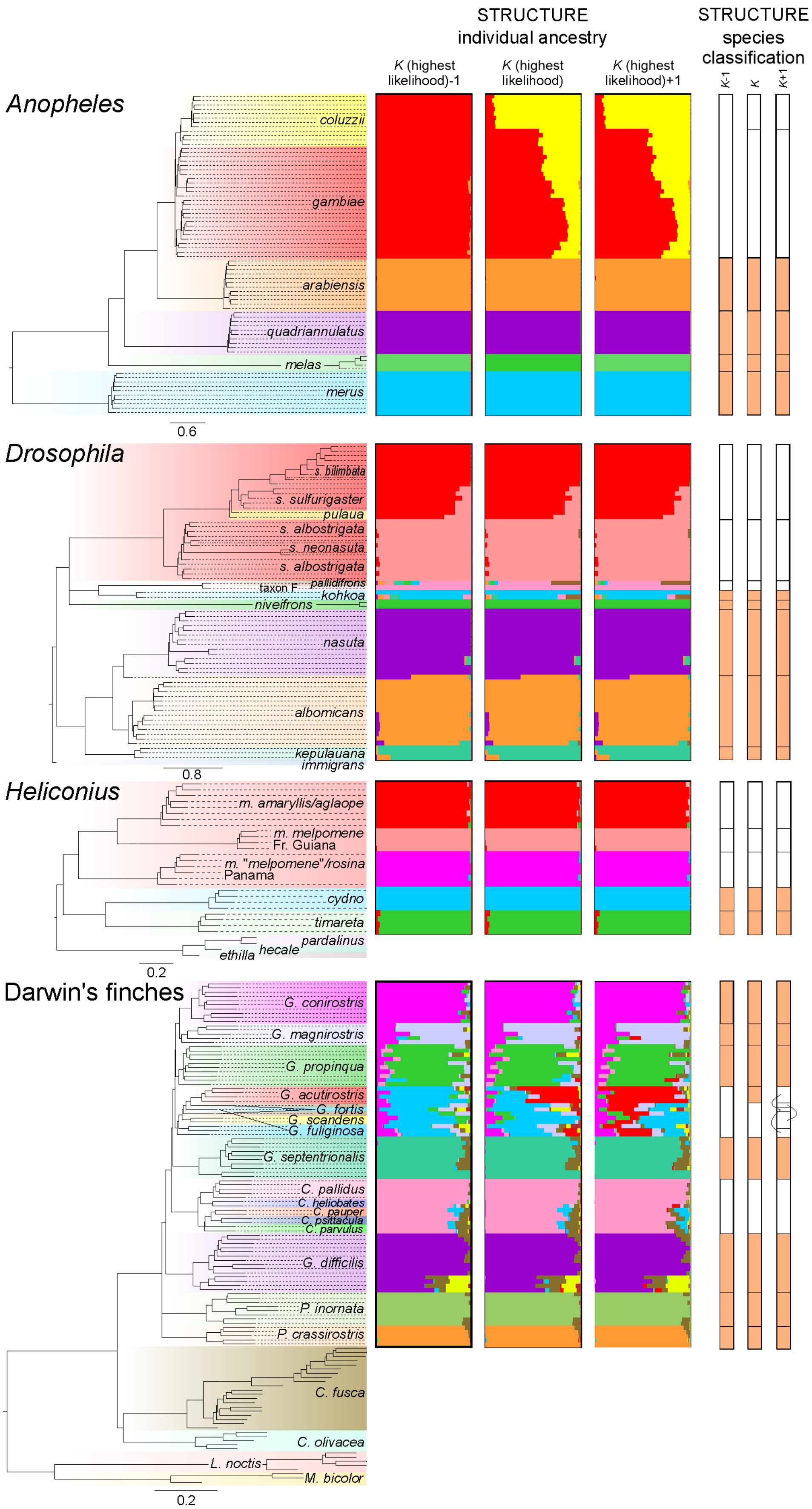

MOLECULAR ECOLOGY

TABLE S1 Comparison of species classifications derived from the STRUCTURE results calculated with three different *K* based on the BUSCO dataset. Numbers in parentheses refer to modified classifications suggested in the text.

	K	Number of species	Number of individuals	Species delimited	Species delimited in accordance with the current classification	Species delimited in accordance with the current classification (%)	Individuals assigned to the same species as in the current classification	Individuals assigned to the same species as in the current classification (%)
Anopheles	8 (run with the highest likelihood)	6	74	6	4(6)	67(100)	70(74)	95(100)
	7	6	74	5	4	67	62(66)	84(89)
	9	6	74	6	4(6)	67(100)	70(74)	95(100)
Drosophila	9 (run with the highest likelihood)	9(8)	67	8	5(8)	56(100)	52(67)	60(100)
	8	9(8)	67	8	5(8)	56(100)	52(67)	60(100)
	10	9(8)	67	8	5(8)	56(100)	52(67)	60(100)
Heliconius	10 (run with the highest likelihood)	3	26	5	2	67	16	62
	9	3	26	5	2	67	16	62
	11	3	26	5	2	67	16	62
Darwin's finches	13 (run with the highest likelihood)	16	87	10	8	50	75	86
	12	16	87	9	7	44	71	82
	14	16	87	10	7	44	73	84
